# Predicting the pathogenicity of novel variants in mitochondrial tRNA with MitoTIP

**DOI:** 10.1371/journal.pcbi.1005867

**Published:** 2017-12-11

**Authors:** Sanjay Sonney, Jeremy Leipzig, Marie T. Lott, Shiping Zhang, Vincent Procaccio, Douglas C. Wallace, Neal Sondheimer

**Affiliations:** 1 Division of Clinical and Metabolic Genetics, The Hospital for Sick Children, Toronto, Ontario, Canada; 2 Department of Biomedical and Health Informatics, The Children’s Hospital of Philadelphia, Philadelphia, Pennsylvania, United States of America; 3 The Center for Mitochondrial and Epigenomic Medicine, The Children’s Hospital of Philadelphia, Philadelphia, Pennsylvania, United States of America; 4 UMR CNRS 6015-INSERM U1083, MitoVasc Institute, Angers University Hospital, Angers, France; 5 Department of Pathology, The University of Pennsylvania, Philadelphia, Pennsylvania, United States of America; 6 Department of Paediatrics, The University of Toronto, Toronto, Ontario, Canada; Universite de Montreal, CANADA

## Abstract

Novel or rare variants in mitochondrial tRNA sequences may be observed after mitochondrial DNA analysis. Determining whether these variants are pathogenic is critical, but confirmation of the effect of a variant on mitochondrial function can be challenging. We have used available databases of benign and pathogenic variants, alignment between diverse tRNAs, structural information and comparative genomics to predict the impact of all possible single-base variants and deletions. The Mitochondrial tRNA Informatics Predictor (MitoTIP) is available through MITOMAP at www.mitomap.org. The source code for MitoTIP is available at www.github.com/sonneysa/MitoTIP.

This is a *PLOS Computational Biology* Software paper.

## Introduction

Variants in mitochondrial tRNAs are an important and common cause of mitochondrial disease. Although some variants have become familiar, determining the pathogenicity of novel identified variants in tRNA-encoding sequences of patients with suspected mitochondrial disease remains problematic. The definitive confirmation of pathogenicity for a novel variant is best accomplished by transmitochondrial cybrid studies [1] or by analysis of heteroplasmy in single muscle fibers [2]. However, both of these studies require laboratory facilities with specialized equipment and specific types of patient samples. As an aid in diagnosis, bioinformatic approaches to predict the effects of variants have been considered previously. Approaches to prediction have used conservation between species [3,4] and machine learning in combination with the presence or absence of heteroplasmy [5]. Here we have predicted the potential impact of all possible variants and single-base deletions from the revised Cambridge Reference Sequence (rCRS) in mitochondrial tRNAs. Our predictive algorithm incorporates an estimation of the importance of a position across all known mitochondrial tRNAs using data from publicly available databases. Comparisons between structurally similar mitochondrial tRNAs improved the sensitivity and specificity of predictions over other available predictive systems.

## Design and implementation

A database of reference benign and pathogenic variants (S1 Table) was created from a comprehensive PubMed search and from publicly available information accessible through MITOMAP [6]. The MITOMAP analysis of sequence diversity is drawn directly from GenBank full sequence data. Interspecific comparison was adapted from Mamit-tRNA and included sequence for all species from the superorder Euarchontoglires [7]. Given the large number of mitochondrial sequences now available from GenBank (n = 37,545 accessed June 2017), we inferred that a lack of observed variation represents a requirement for sequence conservation and we penalized these unobserved variants accordingly. These information sources were combined to provide a **variant history and conservation score** for each variant (see S1 Fig for complete scoring algorithm).

**Fig 1:**
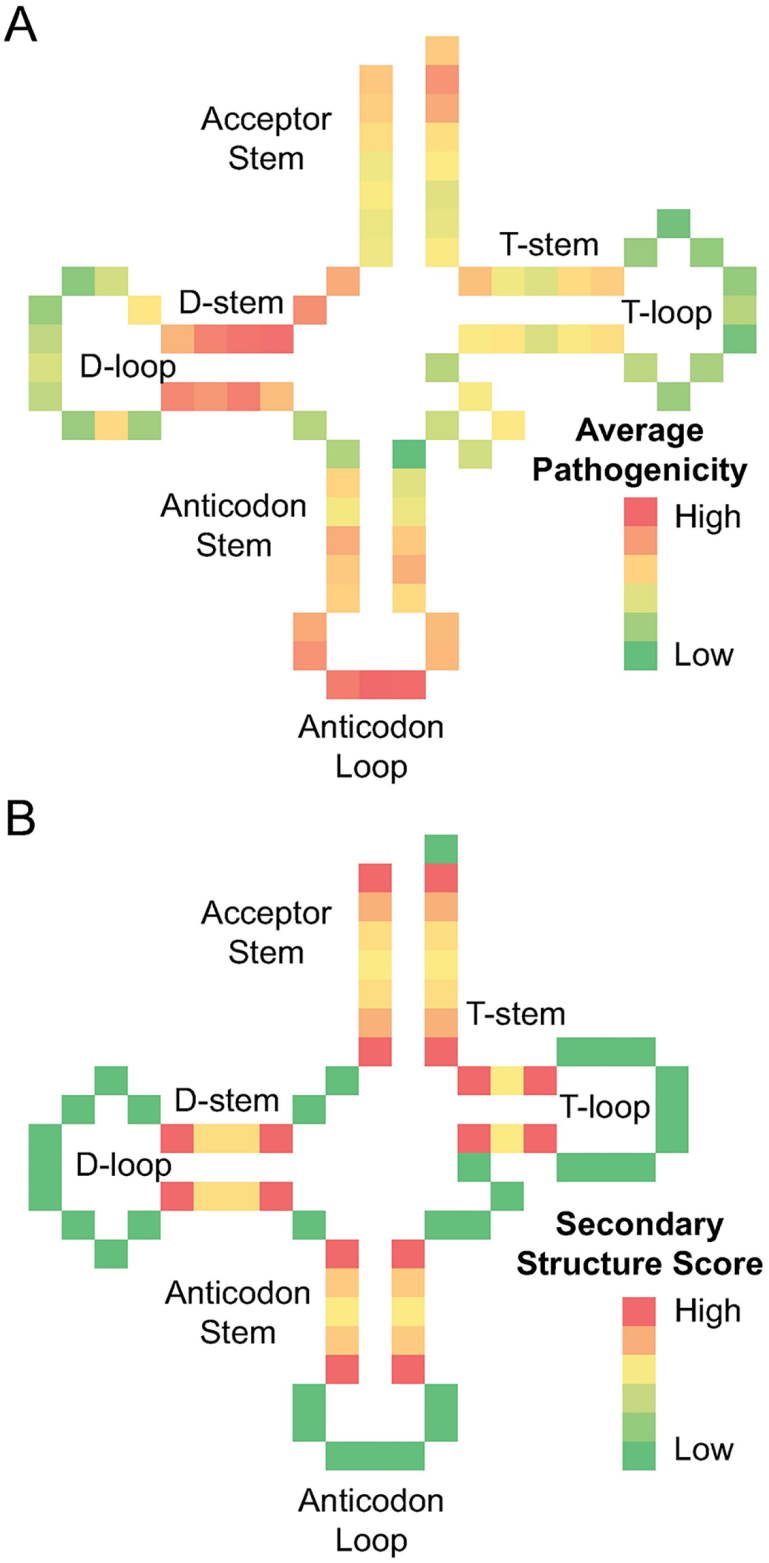
Position and stem penalties for pathogenicity prediction. (A) The variant history and conservation scores at the analogous positions of every mitochondrial tRNA were averaged and used to score a generic tRNA structure. This identifies the regions of the tRNA that are most vulnerable to pathogenic variants. (B) Variants at base pairing regions are assessed based on the steric hindrance they induce, with the highest scores assigned at the ends of the stems region as shown in the scoring heat map for the phenylalanine tRNA.

To create a profile of the likelihood of pathogenic variants occurring at positions within a generic tRNA secondary structure, the sequence of the mitochondrial tRNAs were aligned by anchoring the sequence to the predicted acceptor, D, anticodon and TψC stems, as well as to the anticodon itself. Using this alignment we defined the potential pathogenicity caused by mutation at positions in a generic tRNA (Fig 1A). This sub-scoring, called **position score**, reflected the presence of pathogenic and benign variants in other tRNAs at analogous positions.

Finally, for nucleotides within any of the tRNA stems, we evaluated the steric impact of variants by penalizing for mispairing or bulky substitutions and by penalizing more highly for variants at the end of the stem using a quadratic function (Fig 1B) This sub-scoring was called **secondary structure score**. The total **pathogenicity score** for each possible variant was calculated by summation of the three sub-scores.

## Results and discussion

In order to optimize the system and confirm the validity of pathogenicity prediction we tested it by re-evaluating reference pathogenic (n = 38) and benign (n = 651) variants. To provide an effective test of its ability to discriminate pathogenic and benign variants, we removed available data on each variant iteratively, and examined the ability of the system to predict the effect of each variant naively. The algorithm was modified by altering scaling factors using a differential evolution optimization program (SciPy) that maximized the sensitivity and specificity of the detection system (S2 Table) [8].

**Fig 2:**
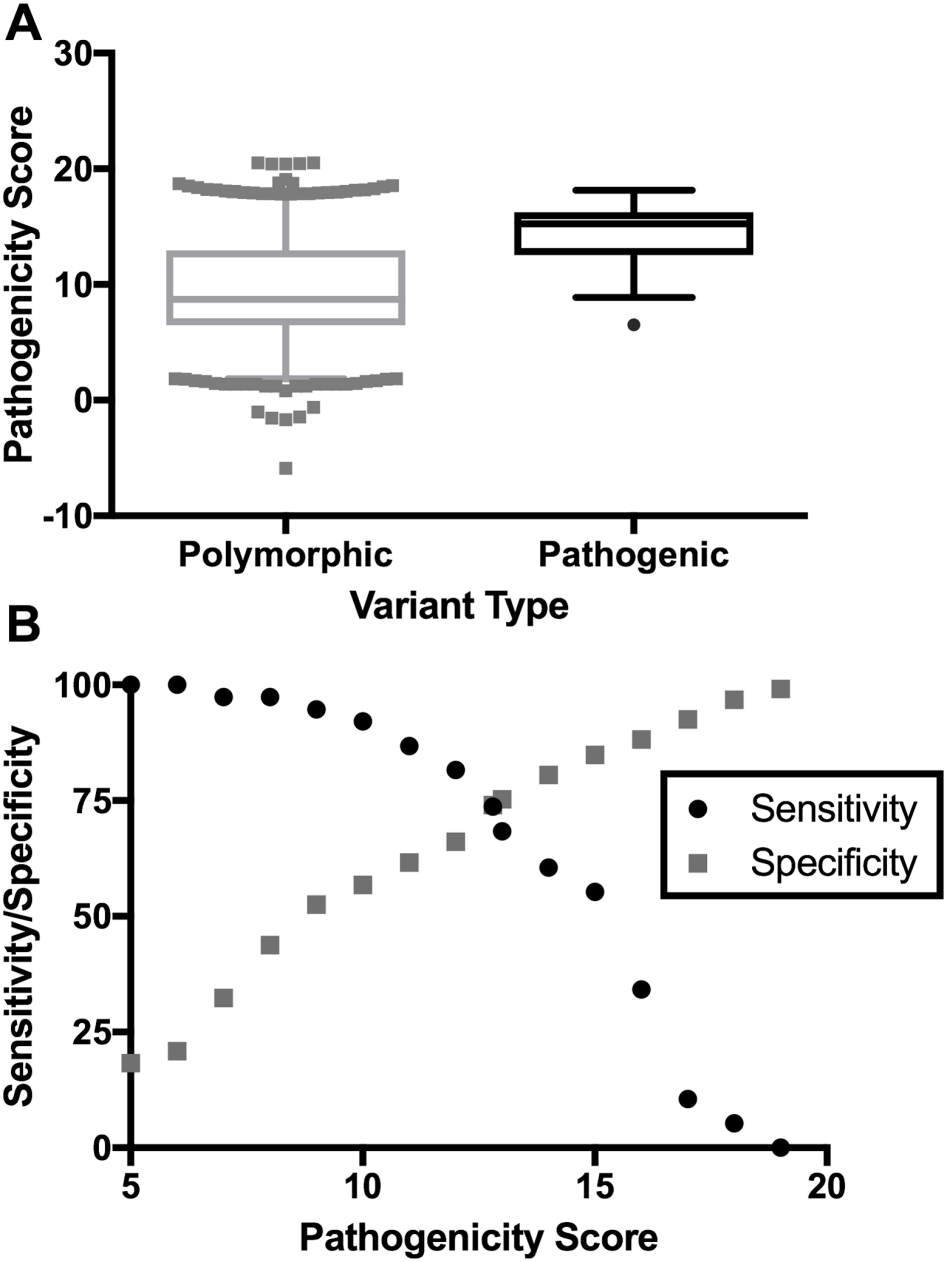
Separation of benign and pathogenic variants by MitoTIP. (A) Pathogenicity scores from naïve evaluations of known pathogenic (n = 38) and described benign (n = 651) variants plotted using box and whiskers at 5–95% (*p*<0.0001 by Mann Whitney test). Negative scoring is possible when polymorphisms improve Watson-Crick pairing in stems. (B) Sensitivity and specificity plot of these data at a range of pathogenicity scores. The crossover pathogenicity score was 12.8.

The final scoring system provided good discrimination of known pathogenic and benign variants (Fig 2A). Using a single point pathogenicity score cutoff, the system had a sensitivity and specificity of 74% (Fig 2B). We compared our system to a more limited set of pathogenic and benign positions that were used to test both the machine learning predictive system and the interspecific homology system proposed by Kondrashov and our system provided superior sensitivity and specificity (Table 1) [3,5].

**Table 1:** Comparison between predictive systems.

System	Sensitivity	Specificity
PON-mt-tRNA	69%	70%
Kondrashov	87%	47%
Mito-TIP	74%	75%

As another demonstration of the specificity of this method, we evaluated scoring at positions associated with haplogroups, as haplogroup-associated variants would generally be presumed to be non-pathogenic. We identified all haplogroup-associated polymorphisms that have been sequenced a minimum of 10 times in GenBank (n = 619). Only three of these exceeded our combined threshold value for pathogenicity, and all of these had been linked to diseases in published studies (see S3 Table). For example, m.5628T>C, which is found in individuals from multiple haplogroups, has been associated with chronic progressive external ophthalmoplegia (CPEO) and hearing loss [9,10]. The reports of pathogenicity in this case are sufficient to inflate the MitoTIP score into the pathogenic range, whereas the same variant analyzed without these reports return a score of possibly benign.

This highlights a disadvantage of MitoTIP’s use of databases. The m.5628T>C variant was initially reported as a heteroplasmic variant with a 40% mutation load causing late-onset CPEO [9]. A second study reports the same variant as being a phenotypic modifier for hearing loss in a family that was homoplasmic for the m.5628T>C variant, but there is no mention of CPEO [10]. This casts doubt on the first study reporting disease association and suggests that this variant may be wrongly classified as pathogenic in both studies.

MitoTIP is designed for the analysis of novel variants, where previous data confirming pathogenicity is unavailable. Several known pathogenic variants such as m.8344A>G score poorly in MitoTIP because the position is neither well conserved nor in a secondary structure location commonly associated with disease. A complete list of the pathogenic mutations scoring in the bottom two quartiles for pathogenicity (n = 5) is provided in S4 Table.

## Availability and future directions

For end users, we have created an interface called the Mitochondrial tRNA Informatics Predictor (MitoTIP—screenshots of interface in S2 Fig). MitoTIP is accessed within the pre-existing structure of MITOMAP (www.mitomap.org). Users can input any tRNA-encoding position into MITOMAP’s point variant search or into MITOMASTER’s SNV Query and retrieve the predicted pathogenicity score of any possible change at that position.

MitoTIP was designed to evaluate novel or infrequently observed single nucleotide variants in tRNA sequence. By design, the display of MitoTIP scoring is suppressed for known pathogenic variants and common variants that are associated with haplogroup. Variants that are confirmed as pathogenic within MITOMAP are listed as “known pathogenic” to avoid confusion. Similarly, high-frequency variants (>1% of all GenBank sequences or >10% in any single major haplogroup division) are listed as “frequent polymorphism.” The use of the MITOMAP platform simultaneously directs users to underlying literature supporting the assignment of variants.

For the target novel mutations, which could all be considered variants of uncertain significance, the pathogenicity prediction is provided by percentile (ranging from 1–99%). Conveniently, the optimal point of the sensitivity/specificity curves is at the 51st centile for pathogenicity scoring. We have chosen to provide an interpretation with four categories (likely pathogenic/possibly pathogenic/possibly benign/likely benign) based upon the quartile scored. We have done this to generally conform with ACMG recommendations for the description of sequence variants [11]. The underlying subpart scoring is also available to interested users.

We have not incorporated the heteroplasmy of a variant into our scoring. It is widely accepted that heteroplasmic variants are more likely to be pathogenic and low-penetrance variants that are homoplasmic are less common. The pathogenicity scoring from MitoTIP for newly observed variants can and should be evaluated by the end user in the context of the actual patient heteroplasmy and the heteroplasmy seen in affected and unaffected family members.

MitoTIP places considerable reliance on databases, which provides important advantages and disadvantages. Full sequence entries used to infer normal human variation might have been obtained from patients with mitochondrial disorders. The underlying calls of pathogenicity represent a best effort at identifying all legitimate reports of mitochondrial variants but may have missed some reports. In addition, pathogenic variant databases may fall out of date or contain errors, as described above for m.5628T>C. The possibility exists that the associations made between haplogroup-defining variants and disease states are incorrect and are due to the coincidence of maternally inherited mitochondrial variants and unmeasured nuclear variants that are actually responsible for the heightened risk of common phenotypes. Providing MitoTIP data in the context of access to these studies will allow users to integrate multiple sources of information when assessing unfamiliar variants.

The use of databases is advantageous because it allows MitoTIP scoring to be easily updated when new information is incorporated into MITOMAP. The system will improve in sensitivity and specificity over time as more sequences are available in MITOMAP and more reports of pathogenic mutations are made.

## Supporting information

S1 Fig Pathogenicity scoring algorithm. The MitoTIP score has three main components: **the variant history and conservation score**, **the position score**, and the **secondary structure score**. The variant history and conservation score is derived from the history of previously reported pathogenic and benign variants, and interspecies sequence conservation. The variant history and conservation data are imported from MITOMAP and Mamit-tRNA, respectively. In benign variants, the GenBank population frequency is calculated and the variants are categorized by percentile rank to generate the **pop score**. Pathogenic variants from the database are stratified by heteroplasmy and whether pathogenicity is confirmed to generate the **path score**. The conservation data for species in the superorder Euarchontoglires was evaluated using a logarithmic function that quantifies each position’s deviation from complete conservation to generate the **cons score**. The **pop score**, **path score**, and **cons score** were evaluated based on the decision tree and scaling factors shown in the figure to generate the **variant hx and conservation score**. The **position score** is calculated by aligning the tRNAs by secondary structure and averaging the **variant history and conservation scores** at the aligned analogous positions. This highlights the positions of the tRNA that are most vulnerable to disease causing variants. Finally the **secondary structure score** is calculated based on the location of the variant within the stem and the steric hindrance induced by the base pair change. Changes at the ends of the stem, and those causing the greatest steric hindrance are considered to be most disruptive to secondary structure and thus assigned the highest scores. Finally the **variant history and conservation score**, **position score**, and **secondary structure score** are scaled by their respective scaling factors and summed to generate the **pathogenicity score**.
(PNG)

S2 Fig MitoTIP interface. (PNG)

S1 Table Variants used in optimization. Pathogenic variants included all variants from MITOMAP with confirmed disease-association plus literature-identified variants meeting the criteria of association with disease and either single-fiber or cybrid confirmation. Benign variants were obtained from the list of “mtDNA Variants” on MITOMAP after filtering out any positions with reports of disease-association.
(DOCX)

S2 Table Optimization of pathogenicity scoring. The MitoTIP algorithm has six scaling factors to adjust the weight of the various sources of information (S1 Fig). The relative weight of variant history (pop and path score) and interspecies conservation (cons score) is represented by the var_hx_scal and cons_scal variables. The weight of the both factors together is scaled by var_hx_cons_scal. The secondary structure score is scaled by the SS_scal variable and the position score is scaled by the Pos_scal variable. Finally, a base_scal variable controls the base score that is applied to novel variants with no previous variant history. In order to optimize these variables we sought to maximize the sensitivity and specificity of MitoTIP at classifying known pathogenic and benign variants (S1 Table) using a take-one-out approach. The SciPy package for python was used to perform differential evolution optimization, which seeks to find the minimum for a multivariate function. The MitoTIP algorithm modified to take the 6 variables as input and output single value that captures the performance of the algorithm. This value was calculated as 2-((sensitivity + specificity)-Abs(sensitivity-specificity)), and is at a minimum when both sensitivity and specificity are maximized. The solution provided by the differential evolution algorithm varies each time that the algorithm is run. The table shows results from four sample runs, with the highlighted row showing the chosen optimized settings for MitoTIP.
(DOCX)

S3 Table Haplogroup defining variants with high pathogenicity scores. (DOCX)

S4 Table Pathogenic variants with low pathogenicity scores. (DOCX)
