# Supplementary figures and images for: Predicting the pathogenicity of novel variants in mitochondrial tRNA with MitoTIP
Source: PLoS Comput Biol. 2017 Dec 11;13(12):e1005867. doi: 10.1371/journal.pcbi.1005867 (PMC5739504; doi:10.1371/journal.pcbi.1005867)

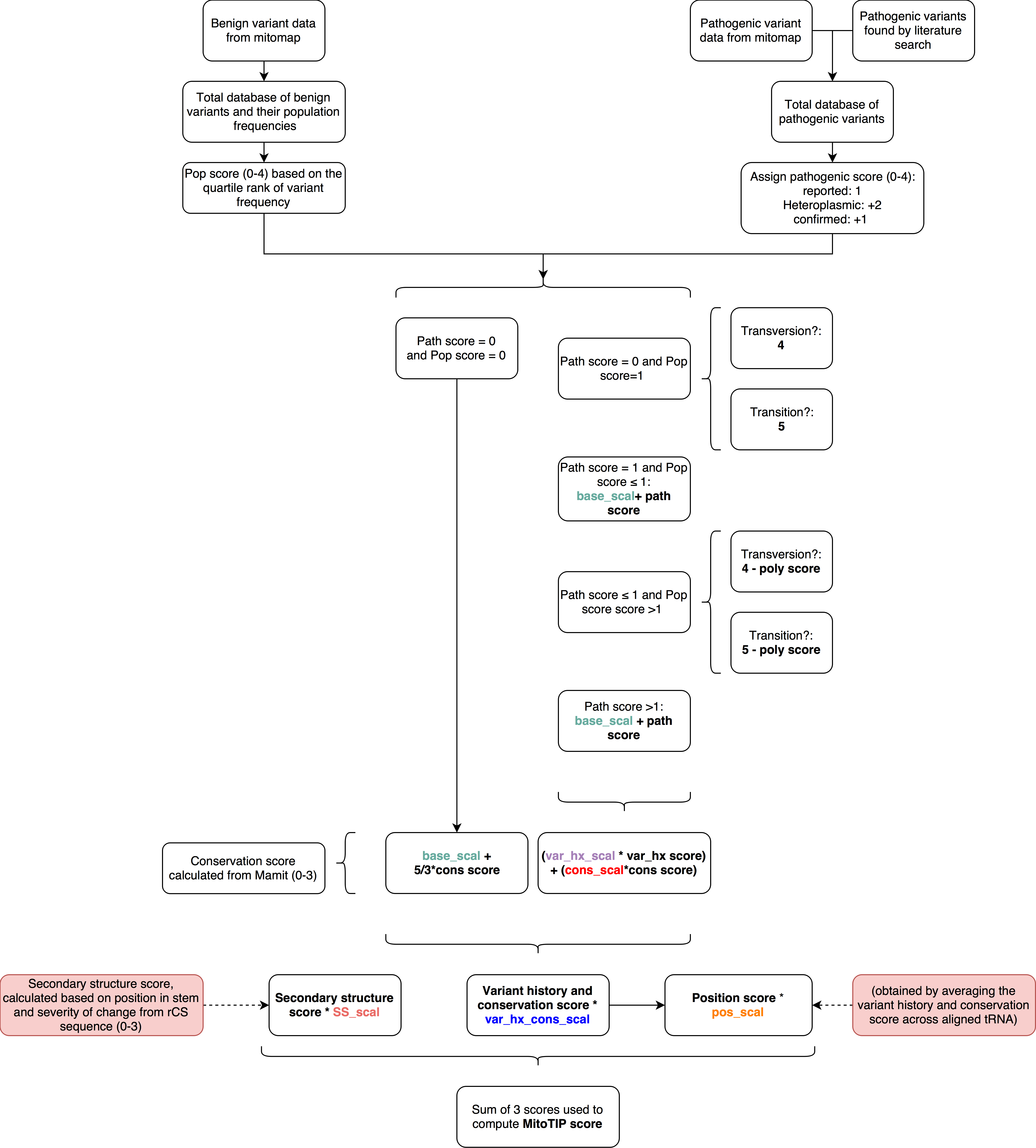

Supplement: S1 Fig — The MitoTIP score has three main components: the variant history and conservation score, the position score, and the secondary structure score. The variant history and conservation score is derived from the history of previously reported pathogenic and benign variants, and interspecies sequence conservation. The variant history and conservation data are imported from MITOMAP and Mamit-tRNA, respectively. In benign variants, the GenBank population frequency is calculated and the variants are categorized by percentile rank to generate the pop score. Pathogenic variants from the database are stratified by heteroplasmy and whether pathogenicity is confirmed to generate the path score. The conservation data for species in the superorder Euarchontoglires was evaluated using a logarithmic function that quantifies each position’s deviation from complete conservation to generate the cons score. The pop score, path score, and cons score were evaluated based on the decision tree and scaling factors shown in the figure to generate the variant hx and conservation score. The position score is calculated by aligning the tRNAs by secondary structure and averaging the variant history and conservation scores at the aligned analogous positions. This highlights the positions of the tRNA that are most vulnerable to disease causing variants. Finally the secondary structure score is calculated based on the location of the variant within the stem and the steric hindrance induced by the base pair change. Changes at the ends of the stem, and those causing the greatest steric hindrance are considered to be most disruptive to secondary structure and thus assigned the highest scores. Finally the variant history and conservation score, position score, and secondary structure score are scaled by their respective scaling factors and summed to generate the pathogenicity score. (PNG) [file pcbi.1005867.s001.png]

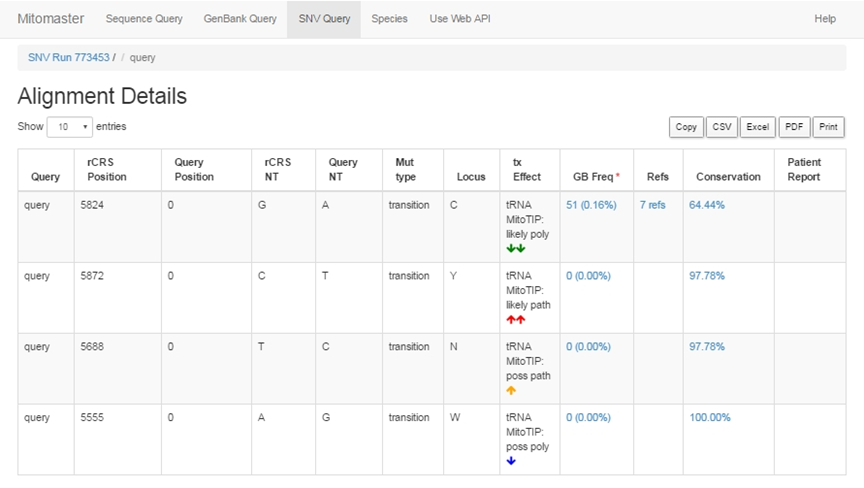

Supplement: S2 Fig — (PNG) [file pcbi.1005867.s002.png]
