# Supplementary material for: Predicting the pathogenicity of novel variants in mitochondrial tRNA with MitoTIP
Source: PLoS Comput Biol. 2017 Dec 11;13(12):e1005867. doi: 10.1371/journal.pcbi.1005867 (PMC5739504; doi:10.1371/journal.pcbi.1005867)
Supplement: S1 Table — Pathogenic variants included all variants from MITOMAP with confirmed disease-association plus literature-identified variants meeting the criteria of association with disease and either single-fiber or cybrid confirmation. Benign variants were obtained from the list of “mtDNA Variants” on MITOMAP after filtering out any positions with reports of disease-association. (DOCX) [file pcbi.1005867.s003.docx]

**S1 Table**

| Pathogenic Variants | Benign Variants | | | | | | | | |
| --- | --- | --- | --- | --- | --- | --- | --- | --- | --- |
| 583A | 580T | 4313T | 5634G | 5875A | 8354T | 12167A | 12312C | 15937C | 16019G |
| 617A | 581C | 4315A | 5639G | 5875T | 8359T | 12170A | 12321G | 15937G | 16020A |
| 1606A | 584A | 4315G | 5640G | 5876G | 8360G | 12170T | 12326G | 15937T | 16020C |
| 1644A | 585A | 4315T | 5641C | 5876T | 9991G | 12171G | 12331G | 15938A | 16020T |
| 3243G | 585G | 4318A | 5646T | 5888T | 9992T | 12172A | 12332G | 15938T | 16021A |
| 3256T | 588T | 4318G | 5651T | 7452G | 9993C | 12172G | 12335G | 15939T | 16021G |
| 3260G | 589A | 4318T | 5653C | 7457C | 9994T | 12173A | 14675G | 15941C | 16021T |
| 3271C | 590A | 4325G | 5654C | 7460G | 9995C | 12173C | 14682G | 15943C | 16022A |
| 3291C | 590C | 4335T | 5662T | 7466C | 9996C | 12174T | 14683G | 15943G | 16022C |
| 3302G | 591A | 4340G | 5663T | 7468T | 10001C | 12175C | 14684T | 15945C | 16022G |
| 3303T | 592A | 4342G | 5664G | 7469T | 10004A | 12175G | 14686A | 15945T | 16022T |
| 4279G | 592C | 4349C | 5668A | 7473G | 10005G | 12176A | 14690G | 15947A |  |
| 4282A | 592T | 4350T | 5671T | 7474A | 10007C | 12178T | 14691T | 15947G |  |
| 4298A | 594A | 4354T | 5673C | 7474G | 10013T | 12179G | 14697T | 15948G |  |
| 4300G | 594T | 4371C | 5674G | 7476T | 10015G | 12180T | 14706G | 15949A |  |
| 4308A | 595A | 4375T | 5675G | 7485C | 10016T | 12181T | 14715A | 15949T |  |
| 4332A | 595C | 4380T | 5675T | 7487T | 10017G | 12182G | 14726G | 15952T |  |
| 4403A | 595T | 4384C | 5681T | 7490G | 10018G | 12184G | 14727C | 15953T |  |
| 5522A | 596C | 4385G | 5683G | 7493T | 10020C | 12186A | 14730T | 15956A |  |
| 5538A | 597C | 4385T | 5686T | 7496C | 10025T | 12188C | 14732G | 15956T |  |
| 5613C | 597T | 4393A | 5700T | 7498A | 10027A | 12189C | 14733A | 15961A |  |
| 5650A | 598A | 4394A | 5701T | 7502C | 10029G | 12190G | 14737C | 15962C |  |
| 5658C | 600A | 4394G | 5704T | 7502T | 10030T | 12191T | 14737G | 15964G |  |
| 5703A | 603G | 4394T | 5705G | 7503C | 10031C | 12193G | 15889C | 15966A |  |
| 5709C | 614G | 4413T | 5711G | 7513T | 10032G | 12194T | 15891C | 15968C |  |
| 7497A | 614T | 4417G | 5713T | 7518G | 10034C | 12195C | 15891G | 15969A |  |
| 7511C | 619C | 4418C | 5715G | 7519A | 10036G | 12196T | 15891T | 15970C |  |
| 7539T | 620T | 4431T | 5764T | 7521A | 10039G | 12200G | 15892C | 15972C |  |
| 8344G | 621G | 4432C | 5765T | 7528G | 10040T | 12210G | 15893C | 15972T |  |
| 8356C | 629A | 4440G | 5768A | 7533T | 10041G | 12212T | 15894A | 15973A |  |
| 8363A | 629C | 4452C | 5769A | 7534T | 10042C | 12215A | 15895C | 15973G |  |
| 10010C | 630T | 4453G | 5771G | 7538G | 10042G | 12215C | 15896G | 15974G |  |
| 12146G | 632T | 4461T | 5772A | 7542G | 10042T | 12216T | 15898C | 15977T |  |
| 12147A | 633G | 4463T | 5773A | 7546C | 10043T | 12217C | 15899C | 15978T |  |
| 12315A | 633T | 4464A | 5774A | 7547C | 10045C | 12217G | 15899T | 15979A |  |
| 14685A | 634A | 4466G | 5774C | 7552G | 10046C | 12218T | 15900C | 15979C |  |
| 14709C | 634C | 4467T | 5774G | 7557G | 10048G | 12223G | 15901G | 15981T |  |
| 14728C | 634G | 4469G | 5775C | 7559G | 10049G | 12231T | 15902C | 15982G |  |
|  | 635T | 5512G | 5776T | 7561C | 10053G | 12234G | 15902G | 15983C |  |
|  | 643T | 5513A | 5777A | 7562G | 10053T | 12235C | 15903C | 15984C |  |
|  | 644G | 5513G | 5779G | 7567T | 10055G | 12237C | 15903G | 15984T |  |
|  | 645A | 5515G | 5782C | 7568C | 10057C | 12237T | 15904A | 15985G |  |
|  | 647G | 5516G | 5785C | 7569A | 10405G | 12238T | 15904T | 15987G |  |
|  | 1603G | 5527G | 5786C | 7569G | 10407T | 12239T | 15905C | 15987T |  |
|  | 1608A | 5528C | 5786T | 7570G | 10409G | 12240T | 15905G | 15989T |  |
|  | 1618G | 5529G | 5788C | 7571C | 10410A | 12241C | 15906C | 15991G |  |
|  | 1619T | 5530T | 5790A | 7571G | 10410C | 12242C | 15906T | 15992G |  |
|  | 1625G | 5539G | 5790T | 7572C | 10411G | 12242G | 15907G | 15992T |  |
|  | 1627A | 5539T | 5793G | 7576G | 10416A | 12245C | 15909C | 15993G |  |
|  | 1629G | 5553C | 5794C | 7579C | 10416C | 12248G | 15909G | 15994G |  |
|  | 1633C | 5553G | 5795A | 7581C | 10420G | 12249T | 15910C | 15996G |  |
|  | 1636G | 5554A | 5797G | 7585A | 10421T | 12250T | 15910T | 15996T |  |
|  | 1646C | 5554G | 5799G | 7585G | 10422G | 12254G | 15911C | 15998C |  |
|  | 1647C | 5554T | 5805G | 8295C | 10423G | 12265G | 15911G | 15999G |  |
|  | 1652A | 5555T | 5806C | 8297C | 10424T | 12266G | 15911T | 16000A |  |
|  | 1654C | 5557C | 5807G | 8297T | 10427A | 12267G | 15912T | 16000C |  |
|  | 1655A | 5558G | 5809A | 8298C | 10428G | 12268C | 15913T | 16000T |  |
|  | 1656A | 5561C | 5810A | 8301G | 10440C | 12273C | 15914C | 16001T |  |
|  | 1656G | 5562C | 5811G | 8307G | 10446G | 12273G | 15914G | 16003C |  |
|  | 1656T | 5563A | 5811T | 8309T | 10448C | 12279G | 15916C | 16004T |  |
|  | 1657T | 5564T | 5812G | 8310C | 10451C | 12279T | 15917C | 16005C |  |
|  | 1661G | 5565G | 5813G | 8312G | 10455G | 12280G | 15917T | 16006G |  |
|  | 1661T | 5566G | 5815T | 8315G | 10456G | 12281G | 15918C | 16006T |  |
|  | 1662T | 5573G | 5817T | 8322C | 10457C | 12281T | 15919T | 16007G |  |
|  | 1664A | 5590A | 5819A | 8323C | 10463A | 12284A | 15921C | 16008C |  |
|  | 1664C | 5595A | 5822A | 8324C | 10463C | 12284T | 15921G | 16010C |  |
|  | 1670T | 5598G | 5823G | 8329G | 10465T | 12285C | 15925T | 16010T |  |
|  | 3248A | 5600G | 5824A | 8331G | 12140G | 12285G | 15926C | 16011A |  |
|  | 3253C | 5603T | 5824T | 8334A | 12141G | 12286G | 15926T | 16011C |  |
|  | 3261G | 5604A | 5826C | 8336C | 12142G | 12290G | 15929G | 16011G |  |
|  | 3281A | 5605G | 5834A | 8338G | 12144G | 12292G | 15930A | 16011T |  |
|  | 3285C | 5606T | 5835A | 8339G | 12151G | 12295C | 15930T | 16013C |  |
|  | 4265G | 5608A | 5836G | 8345T | 12153T | 12298C | 15931T | 16013G |  |
|  | 4266G | 5608T | 5839T | 8346A | 12160G | 12302G | 15932A | 16014A |  |
|  | 4276G | 5615G | 5840T | 8346C | 12161C | 12302T | 15932C | 16016A |  |
|  | 4297G | 5618C | 5841C | 8346T | 12162A | 12303T | 15934G | 16016G |  |
|  | 4301T | 5623A | 5846T | 8349T | 12163G | 12307G | 15935G | 16016T |  |
|  | 4310G | 5631A | 5855G | 8350C | 12164A | 12309G | 15936G | 16017C |  |
|  | 4312T | 5632T | 5864A | 8350G | 12164T | 12310A | 15936T | 16017G |  |
|  | 4313C | 5633T | 5867T | 8351T | 12166C | 12310G | 15937A | 16019A |  |
