# Supplementary material for: Predicting the pathogenicity of novel variants in mitochondrial tRNA with MitoTIP
Source: PLoS Comput Biol. 2017 Dec 11;13(12):e1005867. doi: 10.1371/journal.pcbi.1005867 (PMC5739504; doi:10.1371/journal.pcbi.1005867)
Supplement: S2 Table — The MitoTIP algorithm has six scaling factors to adjust the weight of the various sources of information (S1 Fig). The relative weight of variant history (pop and path score) and interspecies conservation (cons score) is represented by the var_hx_scal and cons_scal variables. The weight of the both factors together is scaled by var_hx_cons_scal. The secondary structure score is scaled by the SS_scal variable and the position score is scaled by the Pos_scal variable. Finally, a base_scal variable controls the base score that is applied to novel variants with no previous variant history. In order to optimize these variables we sought to maximize the sensitivity and specificity of MitoTIP at classifying known pathogenic and benign variants (S1 Table) using a take-one-out approach. The SciPy package for python was used to perform differential evolution optimization, which seeks to find the minimum for a multivariate function. The MitoTIP algorithm modified to take the 6 variables as input and output single value that captures the performance of the algorithm. This value was calculated as 2-((sensitivity + specificity)-Abs(sensitivity-specificity)), and is at a minimum when both sensitivity and specificity are maximized. The solution provided by the differential evolution algorithm varies each time that the algorithm is run. The table shows results from four sample runs, with the highlighted row showing the chosen optimized settings for MitoTIP. (DOCX) [file pcbi.1005867.s004.docx]

**S2 Table**

| Var_hx | Cons  score | SS  score | Var_hx cons score | Pos  score | Base  score | Threshold | Sensitivity | Specificity |
| --- | --- | --- | --- | --- | --- | --- | --- | --- |
| 8.2 | 1.8 | 4.0 | 7.0 | 10.2 | 2.1 | 11.4 | 71.8% | 70.5% |
| 4.2 | 5.8 | 7.4 | 7.0 | 9.5 | 2.2 | 12.3 | 69.2% | 68.8% |
| 6.6 | 3.4 | 8.5 | 7.8 | 9.8 | 2.9 | 13.7 | 69.2% | 70.2% |
| 8.6 | 1.4 | 7.9 | 11.5 | 3.0 | 4.1 | 12.8 | 74.4% | 74.0% |
