# Supplementary material for: Predicting the pathogenicity of novel variants in mitochondrial tRNA with MitoTIP
Source: PLoS Comput Biol. 2017 Dec 11;13(12):e1005867. doi: 10.1371/journal.pcbi.1005867 (PMC5739504; doi:10.1371/journal.pcbi.1005867)
Supplement: S3 Table — (DOCX) [file pcbi.1005867.s005.docx]

**S3 Table**

| **Variant** | **Haplogroup** | **Sequenced** | **Pathogenicity** | **Association** |
| --- | --- | --- | --- | --- |
| m.5628T>C | F1d | 13 | 16.7 | CPEO / DEAF enhancer |
| m.5783G>A | M3a | 20 | 15.0 | Myopathy deafness |
| m.3254C>A | L2 | 18 | 13.8 | Gestational Diabetes |

CPEO: Chronic Progressive External Ophthalmoplegia
