# Supplementary material for: Predicting the pathogenicity of novel variants in mitochondrial tRNA with MitoTIP
Source: PLoS Comput Biol. 2017 Dec 11;13(12):e1005867. doi: 10.1371/journal.pcbi.1005867 (PMC5739504; doi:10.1371/journal.pcbi.1005867)
Supplement: S4 Table — (DOCX) [file pcbi.1005867.s006.docx]

**S4 Table**

| Variant | MitoTIP Score | Phenotype |
| --- | --- | --- |
| 8344G | 11.25 | Myoclonic Epilepsy with Ragged Red Fibers |
| 3291C | 12.10 | Mitochondrial Encephalomyopathy, Lactic Acidosis and Stroke-Like Episodes (MELAS) |
| 1644A | 12.59 | Hypertrophic Cardiomyopathy and MELAS |
| 14728C | 12.61 | Late-onset encephalomyopathy |
| 5709C | 12.63 | Progressive External Ophthalmoplegia |
